# Effect of Hydrogen Peroxide on the Biosynthesis of Heme and Proteins: Potential Implications for the Partitioning of Glu-tRNA^Glu^ between These Pathways

**DOI:** 10.3390/ijms151223011

**Published:** 2014-12-11

**Authors:** Carolina Farah, Gloria Levicán, Michael Ibba, Omar Orellana

**Affiliations:** 1 Programa de Biología Celular y Molecular, Instituto de Ciencias Biomédicas, Facultad de Medicina, Universidad de Chile, Santiago 8380453, Chile; 2 Departamento de Biología, Facultad de Química y Biología, Universidad de Santiago de Chile, Santiago 9170022, Chile; E-Mail: gloria.levican@usach.cl; 3 Department of Microbiology and Center for RNA Biology, Ohio State University, Columbus, OH 43210-1292, USA; E-Mail: ibba.1@osu.edu

**Keywords:** heme, oxidative stress, acidophilic, aminoacyl-tRNA

## Abstract

Glutamyl-tRNA (Glu-tRNA^Glu^) is the common substrate for both protein translation and heme biosynthesis via the C_5_ pathway. Under normal conditions, an adequate supply of this aminoacyl-tRNA is available to both pathways. However, under certain circumstances, Glu-tRNA^Glu^ can become scarce, resulting in competition between the two pathways for this aminoacyl-tRNA. In *Acidithiobacillus ferrooxidans*, glutamyl-tRNA synthetase 1 (GluRS1) is the main enzyme that synthesizes Glu-tRNA^Glu^. Previous studies have shown that GluRS1 is inactivated *in vitro* by hydrogen peroxide (H_2_O_2_). This raises the question as to whether H_2_O_2_ negatively affects *in vivo* GluRS1 activity in *A. ferrooxidans* and whether Glu-tRNA^Glu^ distribution between the heme and protein biosynthesis processes may be affected by these conditions. To address this issue, we measured GluRS1 activity. We determined that GluRS1 is inactivated when cells are exposed to H_2_O_2_, with a concomitant reduction in intracellular heme level. The effects of H_2_O_2_ on the activity of purified glutamyl-tRNA reductase (GluTR), the key enzyme for heme biosynthesis, and on the elongation factor Tu (EF-Tu) were also measured. While exposing purified GluTR, the first enzyme of heme biosynthesis, to H_2_O_2_ resulted in its inactivation, the binding of glutamyl-tRNA to EF-Tu was not affected. Taken together, these data suggest that in *A. ferrooxidans*, the flow of glutamyl-tRNA is diverted from heme biosynthesis towards protein synthesis under oxidative stress conditions.

## 1. Introduction

Heme is a fundamental molecule for living organisms, as the cofactor for several proteins and enzymes involved in cellular processes, such as transport of gases, redox reactions and electron transport [1,2]. Despite its significant roles, heme synthesis must be carefully balanced due to the potentially toxic effects of both heme itself and its intermediates [3,4,5,6]. Heme synthesis occurs by a universally conserved metabolic pathway for synthesizing tetrapyrroles, which starts with δ-aminolevulinic acid (ALA) [1,2]. There are two ways to synthesize ALA. While animals, fungi and α-proteobacteria use the Shemin pathway, plants, archaea and most bacteria use the C_5_ pathway [2]. In the Shemin pathway, ALA is formed from the condensation of glycine and succinyl-CoA, a reaction that is catalyzed by ALA synthase (ALAS). In the C_5_ pathway, ALA is synthesized from Glu-tRNA^Glu^ in two steps. First, the glutamate moiety of Glu-tRNA^Glu^ is reduced to glutamate semialdehyde (GSA) by glutamyl-tRNA reductase (GluTR), and then GSA is converted to ALA by the glutamate semialdehyde 1-2 aminomutase (GSAM) [1,2]. In organisms that use the C_5_ pathway, Glu-tRNA^Glu^ is a common substrate for heme and protein synthesis and must be partitioned between these two processes [7,8]. This distribution is likely determined at least partially by the demand for Glu-tRNA^Glu^ from each pathway, which in turn depends on the physiology of each particular organism. In chemolithoautotrophic bacteria like *Acidithiobacillus ferrooxidans* that use the C_5_ pathway to synthesize tetrapyrroles, high demand for Glu-tRNA^Glu^ for heme biosynthesis is expected, due to the high cytochrome content required for respiration using poor electron donors, such as ferrous ions [9,10]. This bacterium has a complex system of glutamyl-tRNA formation composed of two non-discriminating glutamyl-tRNA synthetases (GluRS1 and GluRS2) and up to four tRNA^Glu^ isoacceptors, with GluRS1 serving as the main enzyme for Glu-tRNA^Glu^ formation [9,11,12]. Three out of four glutamyl-tRNAs can act as donors for both heme and protein synthesis, while the fourth is not a substrate of GluTR and likely acts exclusively in protein synthesis [12]. The above underscores the importance of maintaining a balanced Glu-tRNA^Glu^ supply to each of these metabolic pathways in this organism. Under conditions that reduce the supply of Glu-tRNA^Glu^, both heme and protein synthesis could be compromised. Thus, increased competition between heme and protein biosynthesis pathways for this substrate might change the distribution of Glu-tRNA^Glu^ toward one process or the other. Since it is known that *A. ferrooxidans* GluRS1 is inactivated *in vitro* by H_2_O_2_ [13], we wondered whether inactivating this enzyme might also occur *in vivo*, potentially lowering the pool of Glu-tRNA^Glu^ in the cell and affecting the partitioning of Glu-tRNA^Glu^ towards heme *vs.* protein synthesis. To investigate this question, we determined the effect of H_2_O_2_ on GluRS1 activity and heme content in *A. ferrooxidans*. We found that treating *A. ferrooxidans* with H_2_O_2_ effectively decreased the intracellular activity of GluRS1 and correlated with reduced intracellular heme levels. To provide insight into the fate of Glu-tRNA^Glu^ under this condition, we determined the *in vitro* effect of H_2_O_2_ on the deacylation of Glu-tRNA^Glu^ by GluTR and the binding of Glu-tRNA^Glu^ to elongation factor Tu (EF-Tu). We observed that upon treatment with H_2_O_2_, the activity of purified GluTR, as well as its activation by GSAM, was reduced, while the binding of Glu-tRNA^Glu^ to EF-Tu was not affected. Whether these effects of H_2_O_2_ on GluTR and EF-Tu occur *in vivo* is yet to be determined. We speculate that oxidative stress may reduce intracellular Glu-tRNA^Glu^ concentration, potentially further reducing the partition of this aminoacyl-tRNA towards heme synthesis. Conversely, distribution of the substrate towards protein synthesis might be less affected.

## 2. Results and Discussion

### 2.1. Hydrogen Peroxide Inactivates Glutamyl-tRNA Synthetase 1 (GluRS1) and Reduces Heme Levels in A. ferrooxidans

**Figure 1:**
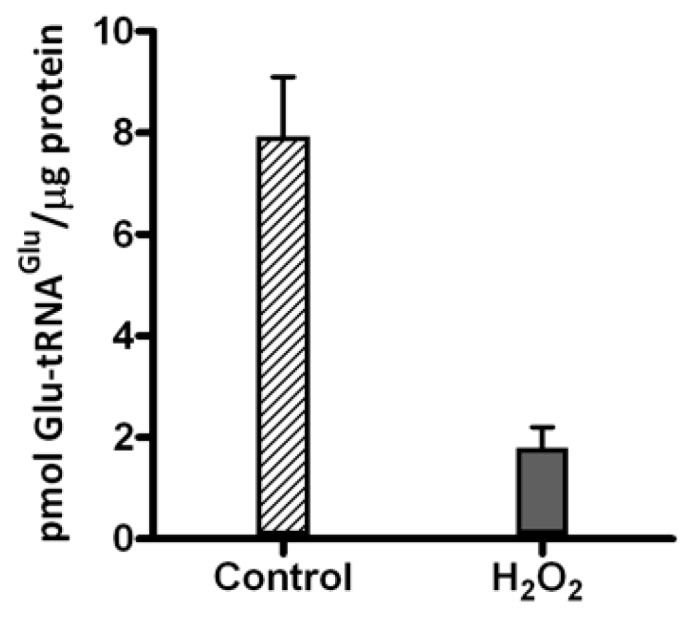
Glutamyl-tRNA synthetase 1 (GluRS1) activity in *A. ferrooxidans* extracts. Specific activity of GluRS1 in extracts from *A. ferrooxidans* treated with 1 mM H_2_O_2_ for 30 min was measured. Control extract was prepared from cells not treated with H_2_O_2_. The mean ± SD values for pmol of Glu-tRNA_2_^Glu^/µg protein formed after 15 min in three different extracts for each condition are shown.

Given that GluRS1 is inactivated by H_2_O_2_ *in vitro* [13], we sought to determine whether GluRS1 activity is decreased *in vivo* in *A. ferrooxidans* exposed to H_2_O_2_. Cellular extracts from *A. ferrooxidans* treated with 1 mM H_2_O_2_ were obtained, and the specific activity of GluRS1 was evaluated in these extracts by the aminoacylation of tRNA_2_^Glu^, a specific substrate of GluRS1 [9,11]. Glu-tRNA_2_^Glu^ formation was decreased by around 80% in this extract relative to the control (Figure 1). Thus, we can conclude that H_2_O_2_ also inactivates GluRS1 *in vivo*. Along with inactivating GluRS1, treating *A. ferrooxidans* with H_2_O_2_ also affected heme levels. After treating the cells with H_2_O_2_ for 2.5 h, intracellular heme concentration fell from 17.2 ± 0.75 pmol/µg of protein in control cells to 12.9 ± 0.23 pmol/µg. Inactivating GluRS1 by H_2_O_2_ exposure, then may reduce intracellular availability of Glu-tRNA^Glu^. Under such conditions, competition between the heme and protein biosynthesis pathways for Glu-tRNA^Glu^ may increase. As a first step to test this hypothesis, we evaluated the effect of H_2_O_2_ on the deacylation of Glu-tRNA^Glu^ by GluTR and its binding to EF-Tu (see below).

### 2.2. Glutamyl-tRNA Reductase (GluTR) from A. ferrooxidans Is Inactivated by Hydrogen Peroxide

**Figure 2:**
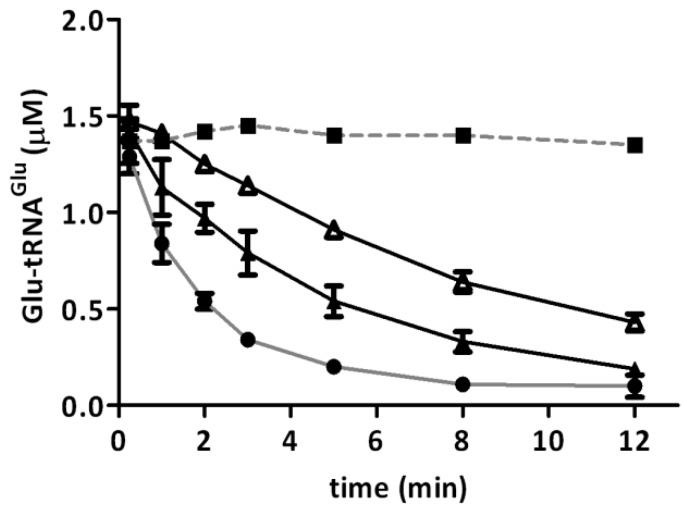
Inactivation of glutamyl-tRNA reductase (GluTR) by H_2_O_2_. Recombinant purified GluTR from *A. ferrooxidans* was incubated with H_2_O_2_ for 20 min at 37 °C. After treatment, activity was measured as the depletion of Glu-tRNA_2_^Glu^ by GluTR (1 µM) that was untreated (**●**) or treated with 250 μM (▲) or 500 μM H_2_O_2_ (Δ). Black squares (■) represent the control without enzyme. Each curve represents the mean of three independent determinations. For each point, standard deviation was no higher than 13%.

GluTR is the first enzyme committed to the biosynthesis of heme and is a key regulatory enzyme of the C_5_ pathway [14,15,16]. Therefore, in order to determine whether Glu-tRNA^Glu^ is diverted from heme biosynthesis under experimental conditions, we determined the effect of H_2_O_2_ on the activity of the recombinant purified enzyme. The deacylation of Glu-tRNA_2_^Glu^ catalyzed by GluTR was used to measure the enzymatic activity. We found that the presence of H_2_O_2_ decreased GluTR activity (Figure 2). Additionally, we observed that higher levels of heme bound to *A. ferrooxidans* GluTR [17] enhanced inactivation by H_2_O_2_. Enzyme preparations with one molecule of heme bound per four GluTR subunits (heme/protein ratio of 1/4) had an increased inactivation rate compared to enzymes with one heme per twelve GluTR subunits (heme/protein ratio of 1/12) (Table 1).

**Table 1:** Inactivation rates (*k*_obs_) of GluTR by H_2_O_2_. Numbers in parentheses represent the relative standard deviation of three experimental replications. *k*_obs_ is the first-order inactivation rate obtained from the slope of ln *N*/*N*_0_ against time. For detailed procedures, see Materials and Methods (GluTR Activity).

Type of GluTR (H_2_O_2_)	GluTR (1/4) 250 µM H_2_O_2_	GluTR (1/4) 500 µM H_2_O_2_	GluTR (1/12) 500 µM H_2_O_2_
*k*_obs_ (s^−1^)	3.18 × 10^−4^ ± (4.43 × 10^−5^)	6.96 × 10^−4^ ± (4.49 × 10^−5^)	4.82 × 10^−4^ ± (2.29 × 10^−5^)

### 2.3. Hydrogen Peroxide Decreases the Stimulation of GluTR by Glutamate Semialdehyde 1-2 Aminomutase (GSAM)

**Figure 3:**
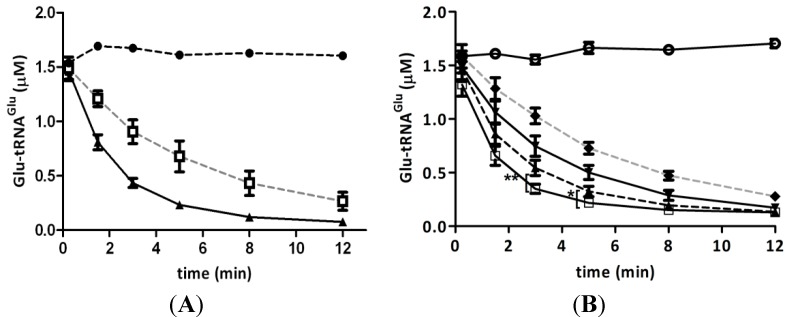
Stimulation of GluTR by glutamate semialdehyde 1-2 aminomutase (GSAM). (**A**) The effect of GSAM on GluTR activity was analyzed by determining the deacylation of Glu-tRNA_2_^Glu^ catalyzed by GluTR (1 µM heme/protein ratio of 1/12) alone (**□**) or in the presence of GSAM (▲). Black circles (●) correspond to deacylation of Glu-tRNA_2_^Glu^ without enzyme; (**B**) The effect of H_2_O_2_ on GSAM stimulation of GluTR activity was evaluated by the deacylation of Glu-tRNA_2_^Glu^ catalyzed by GluTR (1 µM, heme/protein ratio of 1/12) in the presence of GSAM when GluTR (▼), GSAM (▲) or both enzymes (♦) were treated with 300 μM H_2_O_2_. Control (**□**) represents enzyme not treated with H_2_O_2_. Open circles (○) correspond to deacylation of Glu-tRNA_2_^Glu^ without enzymes. Asterisks represent statistically significant differences (** *p* = 0.008; * *p* = 0.035).

In *E. coli* and *Chlamydomonas reinhardtii*, GluTR activity is stimulated upon formation of a complex with GSAM [18,19]. As the amount of Glu-tRNA^Glu^ devoted to heme biosynthesis depends on GluTR activity, we evaluated whether recombinant GluTR activity is stimulated by recombinant *A. ferrooxidans* GSAM. We found that GSAM effectively stimulated GluTR activity (Figure 3A). This effect was observed only when GluTR contained a heme/protein ratio of 1/12. To further investigate whether H_2_O_2_ affected the stimulation of GluTR by GSAM, the deacylation of Glu-tRNA_2_^Glu^ was assayed after treating these enzymes with H_2_O_2_. As shown in Figure 3B, the stimulation of GluTR by GSAM was reduced when GSAM was treated with H_2_O_2_. These data, in turn, indicated that both GluTR activity and the stimulation of this enzyme by GSAM were reduced by treatment with H_2_O_2_. If these effects take place *in vivo*, a reduced partitioning of Glu-tRNA^Glu^ to heme biosynthesis might occur after treating *A. ferrooxidans* with H_2_O_2_. These results are consistent with the reduced heme levels observed in cells treated with H_2_O_2_ (see above).

### 2.4. Elongation Factor Tu (EF-Tu) Is not Affected by Treatment with H_2_O_2_

**Figure 4:**
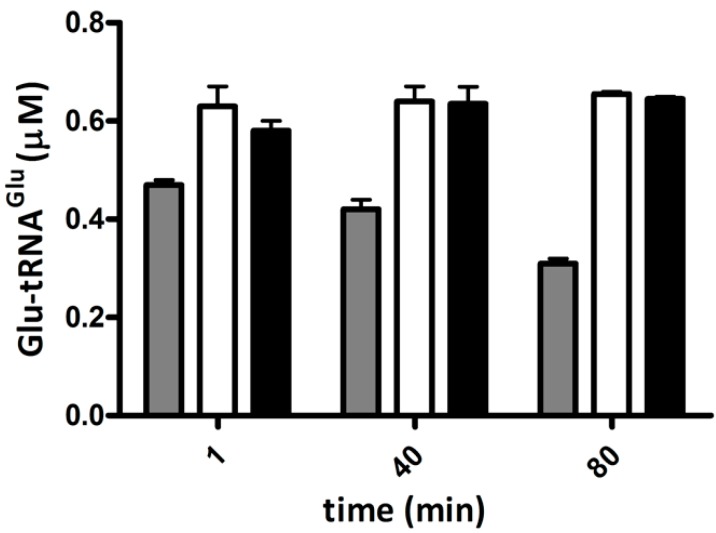
Protection of Glu-tRNA_2_^Glu^ by elongation factor Tu (EF-Tu) against deacylation. Remaining Glu-tRNA_2_^Glu^ over time was determined in the presence of EF-Tu, treated with 500 μM H_2_O_2_ (black bars) or untreated (open bars) at 37 °C. The spontaneous deacylation of Glu-tRNA_2_^Glu^ without EF-Tu was use as a control (gray bars).

The amount of Glu-tRNA^Glu^ dedicated to protein synthesis depends in part on its binding to EF-Tu. As a measure of Glu-tRNA^Glu^ binding to EF-Tu, we assessed the protection of Glu-tRNA^Glu^ by EF-Tu against spontaneous deacylation. Purified recombinant EF-Tu from *A. ferrooxidans* was incubated with H_2_O_2_, and then the ability of this protein to protect Glu-tRNA^Glu^ from spontaneous deacylation was tested. EF-Tu treated with H_2_O_2_ was still able to protect Glu-tRNA^Glu^ from deacylation (Figure 4), implying that the ability of EF-Tu to bind Glu-tRNA^Glu^ was not altered by H_2_O_2_.

**Figure 5:**
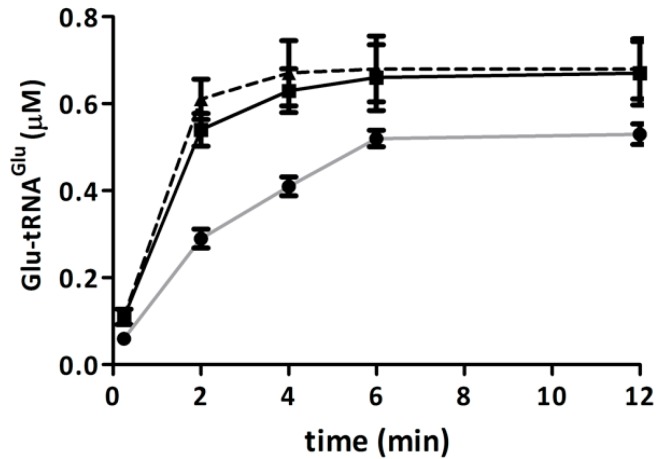
Activity of GluRS1 in presence of EF-Tu. The aminoacylation of tRNA_2_^Glu^ catalyzed by GluRS1 was determined in the absence (●) or presence of EF-Tu, treated with 500 μM H_2_O_2_ (▲) or untreated (■).

Since class I aminoacyl-tRNA synthetases are stimulated by EF-Tu [20], and this stimulation depends on the binding of the aminoacyl-tRNA to EF-Tu, we measured the effect of H_2_O_2_ on the activation of purified recombinant GluRS1 by purified recombinant EF-Tu. We observed that EF-Tu exposed to H_2_O_2_ retained its ability to stimulate GluRS1 activity (Figure 5). Taken together, the data suggest that treating recombinant purified EF-Tu with H_2_O_2_ does not affect the binding of Glu-tRNA^Glu^.

### 2.5. Competition of GluTR and EF-Tu for Glu-tRNA^Glu^ Is Affected by H_2_O_2_

**Figure 6:**
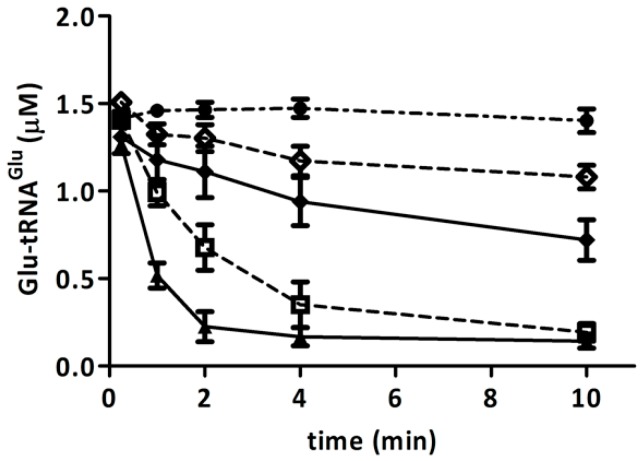
Competition between GluTR and EF-Tu for Glu-tRNA^Glu^. The deacylation of Glu-tRNA_2_^Glu^ catalyzed by GluTR (0.5 µM) in presence of EF-Tu is shown. Deacylation was monitored when GluTR was untreated (▲,♦) or previously treated (**□**,◊) with 300 μM H_2_O_2_ and in the presence of EF-Tu, treated (◊) or untreated (♦) with 300 μM H_2_O_2_. The deacylation of Glu-tRNA_2_^Glu^ in the absence of GluTR or EF-Tu was used as a control (**●**). Each curve is the mean of three experimental replications. For each point, the standard deviation was no higher than 15%.

The observation that, after treatment with H_2_O_2_, recombinant GluTR was inactivated while recombinant EF-Tu maintained its ability to bind Glu-tRNA^Glu^, led us to test to whether EF-Tu would compete with GluTR for Glu-tRNA^Glu^. We evaluated the protection of Glu-tRNA^Glu^ by EF-Tu against the deacylation catalyzed by GluTR. We found that EF-Tu effectively protected Glu-tRNA^Glu^ from deacylation by GluTR, indicating that GluTR and EF-Tu compete for Glu-tRNA^Glu^ (Figure 6). Furthermore, when GluTR and EF-Tu were both treated with H_2_O_2_, there was increased protection against the deacylation of Glu-tRNA^Glu^ (Figure 6). In the event that this competition takes place *in vivo*, it is possible that under oxidative stress, Glu-tRNA^Glu^ would be diverted from the heme biosynthesis pathway.

### 2.6. Discussion

Glu-tRNA^Glu^ is a shared substrate for the biosynthetic pathways of proteins and porphyrins. Therefore, these pathways likely compete for Glu-tRNA^Glu^. Indeed, it has been described that partial inhibition of protein synthesis increases heme production [7]. This competition might be more pronounced under conditions that affect the availability of Glu-tRNA^Glu^. *A. ferrooxidans* is a chemolithotrophic bacterium with an elevated requirement for Glu-tRNA^Glu^ for the synthesis of heme used in respiration processes. We previously demonstrated that GluRS1, the main enzyme that generates Glu-tRNA^Glu^ for heme and protein synthesis in *A. ferrooxidans*, is inactivated by H_2_O_2_. Here we have shown that endogenous GluRS1 is also inactivated upon treatment of the cells with H_2_O_2_. This inactivation of GluRS1 in cells exposed to H_2_O_2_ might imply a reduction in intracellular Glu-tRNA^Glu^ levels. A concomitant reduction in heme levels in *A. ferrooxidans* after treatment with H_2_O_2_ was also observed. In *E. coli*, reduced GluRS activity was also correlated with decreased Glu-tRNA^Glu^ levels [21]. Reduced Glu-tRNA^Glu^ availability could increase competition for this substrate and thereby affect its partition to heme and protein biosynthetic pathways. Thus, we hypothesize that oxidative stress might enhance competition between these biosynthetic pathways for use of Glu-tRNA^Glu^. Our data using recombinant purified proteins from *A. ferrooxidans* revealed that upon treatment with H_2_O_2_, both GluTR activity and GluTR stimulation by GSAM are reduced. Furthermore, we observed that H_2_O_2_ had no effect on the binding of Glu-tRNA^Glu^ to EF-Tu. When competing for the substrate, treatment of these molecules with H_2_O_2_ favored the binding of Glu-tRNA^Glu^ to EF-Tu over GluTR. Although it remains to be shown empirically, these data lead us to speculate that under oxidative stress, the partitioning of Glu-tRNA^Glu^ to protein translation might be favored over heme biosynthesis. Although increased heme turnover cannot be ruled out, this conjecture correlates well with the lower heme levels in cells treated with H_2_O_2_. Since heme and its precursors generate ROS [3,4,5,6], reduced partitioning of Glu-tRNA^Glu^ to heme biosynthesis in *A. ferrooxidans* could favor cell survival when exposed to oxidative stress. It has been described that in *Cucumis sativus*, the ROS producer methyl viologen decreases ALA synthesis, likely by inactivating GluTR and inhibiting GluTR–GSAM complex formation [22]. Furthermore, other mechanisms to avoid the accumulation of toxic heme intermediates exist. In *Salmonella enteric* and *Rhodobacter sphaeroides*, H_2_O_2_ affects the transcription of genes for heme biosynthesis. In *S. enteric*, H_2_O_2_ decreases the transcription of *hemA*, which encodes GluTR, and induces the expression of *hemH*, which encodes ferrochelatase, the last enzyme in the heme biosynthetic pathway [23]. In *R. sphaeroides*, *hemA* is down-regulated when this bacterium is treated with H_2_O_2_ [24]. In *B. subtilis*, a mutation in *per* increases expression of the operon *hemAXCDBL*, which encodes the enzymes for heme biosynthesis. Secretion of overproduced porphyrins then takes place, preventing toxicity [25]. Thus, different strategies seem to have been developed in nature to overcome the potential negative effects of the accumulation of heme and its intermediates, particularly under oxidative stress conditions.

## 3. Experimental Section

### 3.1. Bacterial Strains and Culture Conditions

*A. ferrooxidans* ATCC 23270 was grown aerobically at 30 °C in a modified 9K medium (0.40 g/L MgSO_4_·7H_2_O, 0.10 g/L (NH_4_)_2_SO_4_, 0.04 g/L K_2_HPO_4_·3H_2_O and 33.3 g/L FeSO_4_). For production and purification of recombinant proteins, *E. coli* BL21 [DE3] or JM109 were grown in Luria–Bertani medium at 37 °C. When necessary, 100 µg/mL ampicillin were added to LB medium.

### 3.2. Overproduction and Purification of Proteins

All recombinant proteins from *A. ferrooxidans* were overexpressed in *E. coli* BL21 [DE3]. Recombinant GluRS and EF-Tu were expressed as GST-fusion proteins and purified by affinity chromatography using glutathione agarose as described [9,12,13,17]. After purification, the Glutathione-*S*-Transferase (GST) tag was removed by digestion with thrombin according to the manufacturer’s instructions. Purification of His6-tagged GluTR and GSAM was carried out with a Ni^2+^ affinity resin column. To obtain recombinant GluTR with reduced heme content, the protein was expressed in *E. coli* BL21 [DE3] in the presence of 100 µM orthophenanthroline before inducing expression with 1 mM isopropyl β-d-1-thiogalactopyranoside (IPTG) [9,17]. Purity of proteins was assessed by polyacrylamide-SDS gel electrophoresis and Coomassie blue staining. One major protein band was observed in all purified proteins except in GSAM, in which one major protein (>80%) of the expected molecular mass and two minor proteins were detected.

### 3.3. Preparation and Purification of tRNA Transcripts

*A. ferrooxidans* tRNA_2_^Glu^ (UUC) was prepared by *in vitro* transcription [26]. The DNA template was prepared by PCR amplification of the tRNA_2_^Glu^ gene cloned in pUC18. The *in vitro* transcription reaction mixture contained 40 mM Tris–HCl pH 8.0, 22 mM MgCl_2_, 2 mM spermidine, 5 mM dithiothreitol (DTT), 0.05 mg/mL bovine serum albumin (BSA), 4 mM each nucleotide triphosphate (NTP), 20 mM guanosine monophosphate (GMP), 1 U pyrophosphatase, 25 ng/µL tRNA_2_^Glu^ and 0.12 ng/µL T7 RNA polymerase. The reaction was incubated for 5 h at 42 °C. Afterwards, the tRNA was purified by anionic exchange using Qiagen-tip100 columns following the recommendations of the manufacturer.

### 3.4. Preparation of A. ferrooxidans Cellular Extracts

*A. ferrooxidans* cells were collected by centrifugation, and the pellet was suspended in 10 mM H_2_SO_4_ and treated with 1 mM H_2_O_2_ for 30 min at 30 °C. After exposure to H_2_O_2_, the cells were washed twice with 10 mM H_2_SO_4_ and suspended in 60 mM HEPES (4-(2-hydroxyethyl)-1-piperazineethanesulfonic acid) pH 8, 300 mM NaCl. The cell suspension was sonicated and the cellular debris removed by centrifugation at 15,000× *g* for 30 min. Next, the supernatant was ultracentrifuged at 150,000× *g* for 90 min to obtain cellular extracts. Proteins were measured using the Bradford procedure and the cellular extracts stored in aliquots at −80 °C until use.

### 3.5. GluRS Activity

The activity of endogenous GluRS1 from *A. ferrooxidans* was measured in cellular extracts with buffer containing 5 mM ATP, 100 µM [14C] Glu, 100 mM HEPES KOH pH 7.2, 30 mM KCl, 12 mM MgCl_2_, 3.7 µg cellular extract and 8.4 µM tRNA_2_^Glu^. The activity of recombinant purified GluRS1 from *A. ferrooxidans* was measured at 37 °C in 100 mM HEPES KOH pH 7.2, 30 mM KCl, 12 mM MgCl_2_, 5 mM ATP, 50 μM [14C] Glu and 0.1 μM GluRS1 [11,13]. The reaction was started by adding 2.6 µM tRNA_2_^Glu^. Aliquots were removed at different times, [14C] Glu-tRNA_2_^Glu^ was precipitated with 15% trichloroacetic acid (TCA), and radioactivity was measured in a scintillation counter. When GluRS1 activity in the presence of EF-Tu was measured, 5 µM EF-Tu treated with or without H_2_O_2_ was included.

### 3.6. GluTR Activity

The activity of purified GluTR was determined by measuring the deacylation of Glu-tRNA_2_^Glu^. Because of the instability of the reaction product glutamate semialdehyde, it is described as the method of choice to assess the activity of this enzyme [9,15,17,22]. Aminoacylated [14C] Glu-tRNA_2_^Glu^ was prepared with GluRS1 using 40 µM [14C] Glu and 10 µM tRNA^Glu^. After preparation, [14C] Glu-tRNA_2_^Glu^ was precipitated and stored at −80 °C. GluTR activity was measured at 37 °C in 30 mM HEPES–KOH pH 7.2, 4 mM KCl, 1.5 mM MgCl_2_, 0.1 µg/µL BSA, 10% glycerol, 2 mM NADPH, 0.5–1.0 μM GluTR and 1–2 µM [14C] Glu-tRNA_2_^Glu^. The reaction was started by adding [14C] Glu-tRNA_2_^Glu^, and at different times aliquots were removed and the remaining [14C] Glu-tRNA_2_^Glu^/mg protein measured after precipitation with 15% TCA. To measure the inactivation rate of GluTR by H_2_O_2_, the reaction was started by adding H_2_O_2_, and at different times, aliquots were taken to determine GluTR activity. The remaining GluTR activity was determined by comparing the enzyme activity of the treated GluTR with the activity of the enzyme under the same conditions without the addition of H_2_O_2_. The remaining activity was plotted as ln *N*/*N*0 against time, where *N*_0_ and *N* are activity at time zero and at a given time, respectively. The slope of the lines obtained represents the first-order inactivation rate (*k*_obs_). Competition assays between GluTR and EF-Tu for Glu-tRNA^Glu^ were carried out by measuring GluTR activity in the presence of 10 µM EF-Tu. The effect of GSAM (9 µM) on GluTR activity was measured by adding 0.1 mM pyridoxal phosphate.

### 3.7. Binding of Glutamyl-tRNA^Glu^ to EF-Tu

To evaluate the binding of EF-Tu to Glu-tRNA, we measured the spontaneous deacylation of [14C] Glu-tRNA_2_^Glu^ in the presence of EF-Tu as described [27,28]. To activate EF-Tu, the protein was incubated at 37 °C for 30 min in a reaction mixture containing 500 mM HEPES–KOH pH 7.2, 200 mM MgCl_2_, 0.8 M NH_4_Cl, 4.3 mM GTP, 3.2 μM phosphoenolpyruvate and 0.15 μM pyruvate kinase. The mixture for measuring the deacylation of Glu-tRNA_2_^Glu^ in the presence of EF-Tu contained 30 mM HEPES–KOH pH 7.2, 4 mM KCl, 1.5 mM MgCl_2_, 0.1 µg/µL BSA, 1 μM Glu-tRNA_2_^Glu^ and 5 μM EF-Tu. The assay was started by adding EF-Tu and was performed at 37 °C.

### 3.8. Protein Oxidation

Treatment of GluTR, EF-Tu and GSAM with H_2_O_2_ was performed at 37 °C for 20 min in 100 mM HEPES–KOH pH 7.2, 30 mM KCl, 12 mM MgCl_2_ and 250–500 µM H_2_O_2_. After treatment, H_2_O_2_ was removed by incubation with 0.1 nM catalase for 5 min at 37 °C.

### 3.9. Heme Measurement

Heme content was measured in the 12,000× *g* supernatant of formic acid extracts from *A. ferrooxidans* as absorbance at 398 nm [29] in an Epoch spectrophotometer (Biotek Instruments Inc., Winooski, VT, USA).

## 4. Conclusions

The data presented here show that H_2_O_2_ reduces heme levels and inactivates GluRS in *A. ferrooxidans* cells. Furthermore, our results concerning the effect of H_2_O_2_ on the enzymatic activity of GluTR, the stimulation exerted on GluTR by GSAM and the binding of Glu-tRNA^Glu^ to EF-Tu allowed us to establish a model in which a limited supply of Glu-tRNA^Glu^ generated by oxidative stress this aminoacyl-tRNA changes its fate, favoring the channeling of Glu-tRNA^Glu^ to the biosynthesis of proteins instead of the biosynthesis of heme. Even though additional experiments *in vivo* are required to probe these statements and we cannot rule out alternative explanations, this could represent a way to avoid the accumulation of heme and its toxic intermediaries, which is more relevant under oxidative stress, when the cellular detoxifying system of oxidizing species is being depleted. In this context, beside the further investigation to establish how *in vivo*, the oxidative stress affects the delivery of Glu-tRNA^Glu^ towards the protein and heme syntheses would be interesting analyzed if the partition of this substrate to the tetrapyrroles biosynthesis is also affected in other organism with high demand of heme under oxidative stress. *Leptospirillum ferriphilum* is an adequate model since it to be characterized by their high heme content [9,10] and share with *A. ferrooxidans* an ecological niche with a high heavy metal content, which generates ROS [30,31,32]. However, both bacteria have different strategies to respond to oxidative stress [33]. While in *A. ferrooxidans* a reduction in the production of ROS seems to be the major response, in *L. ferriphilum* the repairing of damaged molecules takes place [33].

## References

[1] Heinemann I.U., Jahn M., Jahn D. (2008). The biochemistry of heme biosynthesis. Arch. Biochem. Biophys..

[2] Panek H., O’Brian M.R. (2002). A whole genome view of prokaryotic haem biosynthesis. Microbiology.

[3] Sadrzsdeh S., Graf E., Panter S., Hallaway P.E., Eaton J.W. (1984). Hemoglobin: Abiologic fenton reagent. J. Biol. Chem..

[4] Pazos M., Andersen M., Skibsted L.H. (2008). Heme-mediated production of free radicals via preformed lipid hydroperoxide fragmentation. J. Agric. Food Chem..

[5] Hiraku Y., Kawanishi S. (1996). Mechanism of oxidative DNA damage induced by δ-aminolevulinic acid in the presence of copper ion. Cancer Res..

[6] Fotinos N., Convert M., Piffaretti J., Gurny R., Lange N. (2008). Effects on Gram-negative and Gram-positive bacteria mediated by 5-aminolevulinic acid and 5-aminolevulinic acid derivatives. Antimicrob. Agents Chemother..

[7] Nakayashiki T., Nishimura K., Tanaka R., Inokuchi H. (1995). Partial inhibition of protein synthesis accelerates the synthesis of porphyrin in heme-deficient mutants of *Escherichia coli*. Mol. Gen. Genet..

[8] Francklyn C.S., Minajigi A. (2010). tRNA as an active chemical scaffold for diverse chemical transformations. FEBS Lett..

[9] Levicán G., Katz A., de Armas M., Nuñez H., Orellana O. (2007). Regulation of a glutamyl-tRNA synthetase by the heme status. Proc. Natl. Acad. Sci. USA.

[10] Yarzabal A., Brasseur G., Bonnefoy V. (2002). Cytochromes *c* of *Acidithiobacillus ferrooxidans*. FEMS Microbiol. Lett..

[11] Salazar J.C., Ahel I., Orellana O., Tumbula-Hansen D., Krieger R., Daniels L., Söll D. (2003). Coevolution of an aminoacyl-tRNA synthetase with its tRNA substrates. Proc. Natl. Acad. Sci. USA.

[12] Levicán G., Katz A., Valenzuela P., Soll D., Orellana O. (2005). A tRNA^Glu^ that uncouples protein and tetrapyrrole biosynthesis. FEBS Lett..

[13] Katz A., Banerjee R., de Armas M., Ibba M, Orellana O. (2010). Redox status affects the catalytic activity of glutamyl-tRNA synthetase. Biochem. Biophys. Res. Commun..

[14] Li J., Brathwaite O., Cosloy S, Russell C.S. (1989). 5-Aminolevulinic acid synthesis in *Escherichia*
*coli*. J. Bacteriol..

[15] Kang Z., Wang Y., Gu P., Wang Q., Qi Q. (2011). Engineering *Escherichia coli* for efficient production of 5-aminolevulinicacid from glucose. Metab. Eng..

[16] Schobert M., Jahn D. (2002). Regulation of heme biosynthesis in non-phototrophic bacteria. J. Mol. Microbiol. Biotechnol..

[17] De Armas M., Levicán G., Katz A., Moser J., Jahn D., Orellana O. (2011). Cellular levels of heme affect the activity of dimeric glutamyl-tRNA reductase. Biochem. Biophys. Res. Commun..

[18] Luer C., Schauer S., Mobius K., Schulze J., Schubert W., Heinz D.W., Jahn D., Moser J. (2005). Complex Formation between glutamyl-tRNA reductase and glutamate-1-semialdehyde 2,1-aminomutase in *Escherichia coli* during the initial reactions of porphyrin biosynthesis. J. Biol. Chem..

[19] Nogaj L.A., Beale S.I. (2005). Physical and kinetic interactions between glutamyl-tRNA reductase and glutamate-1-semialdehyde aminotransferase of *Chlamydomonas reinhardtii*. J. Biol. Chem..

[20] Zhang C., Perona J., Ryu K., Francklyn C., Hou Y. (2006). Distinct kinetic mechanisms of the two classes of aminoacyl-tRNA synthetases. J. Mol. Biol..

[21] Kaspy I., Rotem E., Weiss N., Ronin I., Balaban N.Q., Glaser G. (2013). HipA-mediated antibiotic persistence via phosphorylation of the glutamyl-tRNA-synthetase. Nat. Commun..

[22] Aarti D., Tanaka R., Ito H., Tanaka A. (2007). High light inhibits chlorophyll biosynthesis at the level of 5-aminolevulinate synthesis during de-etiolation in cucumber (*Cucumis sativus*) cotyledons. Photochem. Photobiol..

[23] Elgrably-Weiss M., Park S., Schlosser-Silverman E., Rosenshine I., Imlay J., Altuvia S. (2002). A *Salmonella enterica* serovar typhimurium *hemA* mutant is highly susceptible to oxidative DNA damage. J. Bacteriol..

[24] Zeller T., Moskvin O.V., Li K., Klug G., Gomelsky M. (2005). Transcriptome and physiological responses to hydrogen peroxide of the facultatively phototrophic bacterium *Rhodobacter sphaeroides*. J. Bacteriol..

[25] Faulkner M.J., Ma Z., Fuangthong M., Helmann J. (2012). Derepression of the *Bacillus subtilis* PerR peroxide stress response leads to iron deficiency. J. Bacteriol..

[26] Sampson J.R., Uhlenbeck O.C. (1988). Biochemical and physical characterization of an unmodified yeast phenylalanine. Proc. Natl. Acad. Sci. USA.

[27] Roy H., Becker H.D., Mazauric M., Kern D. (2007). Structural elements defining elongation factor Tu mediated suppression of codon ambiguity. Nucleic Acids Res..

[28] Ling J., Ran So B., Yadavalli S.S., Roy H., Shoji S., Fredrick K., Musier-Forsyth K., Ibba M. (2000). Resampling and editing of mischarged tRNA prior to translation elongation. Mol. Cell.

[29] Kuross S.A., Rank B.H., Hebbel R.P. (1988). Excess heme in sickle erythrocyte inside-out membranes: Possible role in thiol oxidation. Blood.

[30] Flora J.S., Mittal M., Mehta A. (2008). Heavy metal induced oxidative stress & its possible reversal by chelation therapy. Indian J. Med. Res..

[31] </i>Stohs S.J., Bagchi D. (1995). Oxidative mechanisms in the toxicity of metal ions. Free Radic. Biol. Med..

[32] López-Archilla A.I., Marin I., Amils R. (2001). Microbial community composition and ecology of an acidic aquatic environment: The tinto river, Spain. Microb. Ecol..

[33] Cortés A., Flores R., Norambuena J., Cardenas J.P., Quatrini R., Orellana O., Levicán G., Qiu G., Jiang T., Qin W., Liu X., Yang Y., Wang H. (2011). Comparative study of redox stress response in the acidophilic bacteria *Leptospirillum ferriphilum* and *Acidithiobacillus ferrooxidans*. Biohydrometallurgy, Biotech Key to Unlock Mineral Resources Value.

